# The Role of Formyl Peptide Receptors in Neurological Diseases via Regulating Inflammation

**DOI:** 10.3389/fncel.2021.753832

**Published:** 2021-09-28

**Authors:** Jiahui Zhu, Lingfei Li, Jiao Ding, Jinyu Huang, Anwen Shao, Bo Tang

**Affiliations:** ^1^The Fourth School of Clinical Medicine, Zhejiang Chinese Medical University, Hangzhou, China; ^2^Department of Neurology, The Affiliated Hangzhou First People’s Hospital, Zhejiang University School of Medicine, Hangzhou, China; ^3^Department of Cardiology, The Affiliated Hangzhou First People’s Hospital, Zhejiang University School of Medicine, Hangzhou, China; ^4^Department of Neurosurgery, The Second Affiliated Hospital, School of Medicine, Zhejiang University, Hangzhou, China

**Keywords:** formyl peptide receptors, neuroinflammation, neurodegenerative diseases, neurogenic tumors, cerebrovascular diseases

## Abstract

Formyl peptide receptors (FPRs) are a group of G protein-coupled cell surface receptors that play important roles in host defense and inflammation. Owing to the ubiquitous expression of FPRs throughout different cell types and since they interact with structurally diverse chemotactic agonists, they have a dual function in inflammatory processes, depending on binding with different ligands so that accelerate or inhibit key intracellular kinase-based regulatory pathways. Neuroinflammation is closely associated with the pathogenesis of neurodegenerative diseases, neurogenic tumors and cerebrovascular diseases. From recent studies, it is clear that FPRs are important biomarkers for neurological diseases as they regulate inflammatory responses by monitoring glial activation, accelerating neural differentiation, regulating angiogenesis, and controlling blood brain barrier (BBB) permeability, thereby affecting neurological disease progression. Given the complex mechanisms of neurological diseases and the difficulty of healing, we are eager to find new and effective therapeutic targets. Here, we review recent research about various mechanisms of the effects generated after FPR binding to different ligands, role of FPRs in neuroinflammation as well as the development and prognosis of neurological diseases. We summarize that the FPR family has dual inflammatory functional properties in central nervous system. Emphasizing that FPR2 acts as a key molecule that mediates the active resolution of inflammation, which binds with corresponding receptors to reduce the expression and activation of pro-inflammatory composition, govern the transport of immune cells to inflammatory tissues, and restore the integrity of the BBB. Concurrently, FPR1 is essentially related to angiogenesis, cell proliferation and neurogenesis. Thus, treatment with FPRs-modulation may be effective for neurological diseases.

## Introduction

Formyl peptide receptors (FPRs) (mFPRs in mice) are a small group of G protein-coupled receptors (GPCRs) with seven transmembrane domains. FPRs were initially identified as binding to and activated by N-formyl peptide, which is released from the damaged mitochondria of skin bacteria during tissue injury (Hilger et al., 2018). FPRs transduce chemotactic signals in phagocytes that react to tissue injury-associated chemotactic molecular patterns, which mediate cell adhesion, directed migration, degranulation, and superoxide production, thereby regulating host-defense and inflammatory responses through intracellular calcium mobilization and mitogen-activated protein kinase (MAPK), extracellular-regulated protein kinase (ERK), phosphoinositide-3-kinase (PI3K), protein kinase B (Akt), and p38/MAPK signal transduction pathways (Dorward et al., 2015). Interestingly, FPRs play both anti-inflammatory and pro-inflammatory roles, depending on the ligands type and the inflammatory microenvironment. Increasing attention has been paid to their role in the pathogenesis of both infective and non-infective inflammatory diseases. When FPRs are expressed on leukocytes during bacterial or viral infections, they sense microbe-specific molecular pattern molecules, thereby leading to leukocyte activation and chemotaxis. Additionally, FPRs participate in non-infective inflammation of wound-healing (Liu et al., 2014) and reduce allergic reactions in the airways (de Oliveira et al., 2017), as well as facilitate tumor invasion by regulating tumor-associated inflammation and escape responses (Chen et al., 2017).

Neuroinflammation occurs in the brain and spinal cord, primarily caused by abnormally acute or chronic activation of glial cells. Its intensity and extent varies between the pathogeneses of injury, infection, or stress responses mediated by cytokines, chemokines, and reactive oxygen species (ROS), among secondary messengers (DiSabato et al., 2016). Inflammation causes immune activation, cell death, edema, and tissue damage, which lead to neurological disease. Neuroinflammations regulate neurodegenerative diseases (Alzheimer’s disease), neurological tumors (gliomas, neurilemmas), and cerebrovascular diseases (stroke, carotid stenosis), affecting their occurrence, development and prognosis (DiSabato et al., 2016). The expression and distribution of FPRs in different regions of the brain and spinal cord have been identified (Ho et al., 2018). FPRs in neuronal cells and tissues are significant for the identification of agonists and neural cell expression that trigger multiple intracellular signaling pathways to regulate various pathophysiological conditions (Cussell et al., 2020). The FPR ligands N-formyl-leucine-phenylalanine (fMLF) and serum amyloid A (SAA) exert pro-inflammatory effects, while annexin-1 (AnxA1), lipoxin A4 (LXA4), and resolvin-D1 (RvD1) produce pro-resolving effects. FPRs monitor glial activation (Han et al., 2020), accelerate neural differentiation (Urban et al., 2019), promote axon and dendrite growth (Ho et al., 2018), regulate immune escape of intracranial tumors (Yao et al., 2008), and regulate blood brain barrier (BBB) permeability (Liu H. et al., 2021), thereby affecting the progression of neurological diseases. This review elucidates the recent advances in determining the pluripotent physiological roles of FPRs in the neurological disease microenvironment and proposes several cases that emphasize on the therapeutic potentials of FPRs, especially FPR1 and FPR2, for regulating host defense and fostering innate immunity.

## Formyl Peptide Receptors: Structure, Ligands, and the Signaling Pathway

Formyl peptide receptors belong to the seven-transmembrane GPCR family, many of which play important roles in anti-bacterial host defense mechanisms. Their polypeptide chains are composed of extracellular N-terminal domains, intracellular and extracellular loops, and seven transmembrane helices linked to the intracellular C-terminal. The human FPR family comprises FPR1, FPR2, and FPR3, which are myeloid receptors expressed in monocytes/macrophages and neutrophils, and have phagocytic capabilities (Liu H. et al., 2021). Formylated peptides, non-formylated peptides, synthetic small molecules, and eicosanoids from bacteria and mitochondria can be identified by FPRs to govern inflammatory reactions leading to chemotaxis, degranulation, and oxidative burst (Ye et al., 2009; Schepetkin et al., 2014; Weiß and Kretschmer, 2018). Therefore, FPRs are deemed as pattern-recognition receptors as they interact with both pathogen-associated molecular patterns (PAMPs) and damage-associated molecular patterns (DAMPs) (McDonald et al., 2010; Zhang et al., 2010).

The first-identified and shortest formyl peptide with the potential for full agonistic activity is fMLF, which is derived from *Escherichia coli*. This peptide triggers a variety of biological activities in phagocytes and myelocytes (Marasco et al., 1984). FPRs bind many host-derived non-formyl peptide agonists to regulate pathophysiological processes. Serum amyloid A (SAA) comprises a family of prominent acute-phase proteins, which were the first endogenous peptide agonists identified for FPRs (Su et al., 1999). Recent research shows that SAA exerts type 2 immunity in the respiratory system through the release of IL-33 and induces intestinal epithelial wound closure associated with alterations in the focal adhesion protein p130 CAS localization and ROS induction (Hinrichs et al., 2018; Smole et al., 2020). The pathologies underlying protein misfolding are many, and misfolded proteins are host-derived proteins that induce FPR-activation, particularly FPR2. A familiar amyloidogenic protein associated with an FPR2 agonist is amyloid-β 1-42 (Aβ1-42), a well-documented peptide fragment in patients with Alzheimer’s disease (AD) (Wickstead et al., 2020), and the prion protein fragment PrP (106-126), which interacts with FPRs on astrocytes and microglia and regulates inflammatory process such as calcium mobilization, chemotaxis, and the production of pro-inflammatory cytokines (Le et al., 2001b). AnxA1, LXA4, and RvD1 are regarded as endogenous pro-resolving FPR ligands. AnxA1, also called lipocortin-1, is secreted in response to tissue damage, and its N-terminal peptides, Ac2-26 and Ac9-25, have a concentration-dependent interaction with FPRs (He and Ye, 2017).

An increasing amount of evidence has demonstrated the significant role of AnxA1 in cell proliferation, phagocytosis, apoptosis, and the origination and development of tumors (Foo et al., 2019). LXA4 and RvD1 are host-derived lipid and lipopeptide agonists that impede the activation of FPR2-dependent signaling, including calcium mobilization (He and Ye, 2017). Interestingly, highly phosphorylated FPR3 is localized in small intracellular vesicles, but they are unresponsive to formyl peptides. An acetylated N-terminal fragment of F2L, a human heme-binding protein, and the neuroprotective peptide humanin are characteristic FPR3 agonists. However, the functional role of FPR3 remains unclear (Migeotte et al., 2005; Rabiet et al., 2011).

**FIGURE 1:**
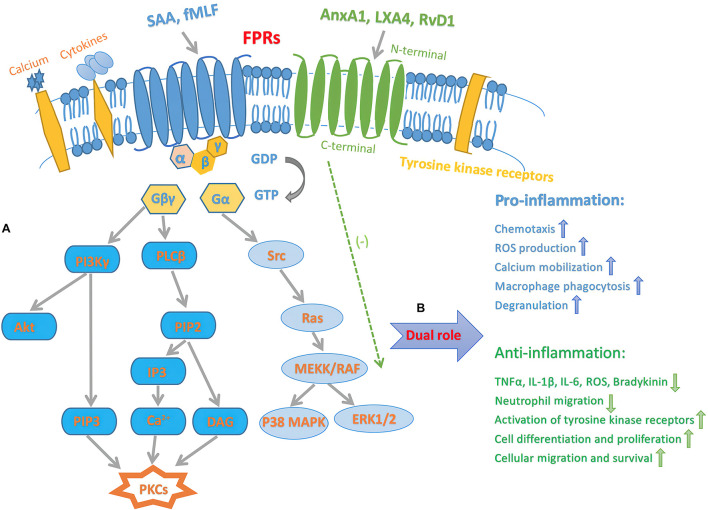
Schematic diagram illustrating inflammatory signaling pathways of FPRs. **(A)** Intracellular transduction signaling pathway of FPRs. The activation of FPRs originates from sensing and binding PAMPs and DAMPs, results in the dissociation of the Gα from the Gβγ subunit. The latter activates PLCβ, resulting in release of calcium from intracellular stores then activate PKCs. Another pathway triggered by the βγ subunit is that PI3Kγ activate PKCs and Akt. Furthermore, α subunit activates GTPases of the Ras superfamily, which further contribute to activation of the MAPK pathways, p38, and ERK1/2. **(B)** Dual pro-inflammatory and pro-resolving effects of FPRs signaling. Endogenous SAA and bacterial original fMLF bind to FPRs, mediate pro-inflammatory actions, such as chemotaxis, superoxide anion production, calcium mobilization, macrophage phagocytosis, and degranulation. On the other hand, FPRs pro-resolving agonists include AnxA1, LXA4, and RvD1. This process prevents producing TNFα, IL-1β, IL-6, ROS, bradykinin and inhibit neutrophil migration. Furthermore, the pro-resolving procedure deactivates tyrosine kinase receptors, leading to downstream signaling event that promote cell differentiation and proliferation, as well as enhancing cellular migration and survival. Gα, G-protein α; Gβγ, G-protein βγ.

The binding of AnxX1, LXA4, RvD1 triggers FPRs activation, leading to signaling pathways that regulate inflammatory responses. Although FPR1 and FPR2 differ in specificity and binding properties, their amino acid sequences are 69% homologous, and therefore, both FPRs are probably activated through the same signaling pathways (Ye et al., 2009). The identification of bacteria-chemotactic PAMPs and host tissue-derived chemotactic DAMPs by FPRs leads to an inflammatory cascade reaction. The FPR ligands, fMLF and SAA exert pro-inflammatory effects, while AnxA1, LXA4, and RvD1 produce pro-resolving effects (Dufton and Perretti, 2010; Bozinovski et al., 2013). The inflammatory response mediated by FPR-signaling pathways contains FPRs regulating pro-inflammatory effects by enhancing leukocyte transport and inhibiting neutrophil apoptosis, whereas receptor-mediated anti-inflammatory effects are also inhibited similarly (Chen et al., 2017). The binding of the ligands to FPR1 or FPR2 causes a conformational change, which subsequently activates downstream molecules. The GTPases of the Ras superfamily, such as Ras, Rho, CDC42, and Rac, activate MAPK pathways, ERK1/2, p38, and JUN-N-terminal protein kinase (JNK) (Zhao et al., 2015). Phospholipase Cβ (PLCβ) is activated by another subunit, leading to the release of intracellular calcium from the endoplasmic reticulum, thereby activating PKC, and ROS production (Kwan et al., 2008; Dorward et al., 2015). Furthermore, the subunit also activates PKCs and Akt. These crucial signal transduction pathways contribute to degranulation, chemotaxis, superoxide production, and transcriptional regulation (Dorward et al., 2015). The recognition of chemotactic PAMPs and DAMPs induces the binding of pro-inflammatory or pro-resolving ligands to FPRs, which may trigger or inhibit a series of intracellular kinase pathways that affect the regulation of various cellular functions (Figure 1).

## Formyl Peptide Receptor Action in Neuroinflammation

Neuroinflammation refers to inflammation that occurs in both central and peripheral nervous system, which involves the glial cells (Liddelow et al., 2017; Yun et al., 2018) and perivascular macrophages (Liddelow et al., 2017; Yun et al., 2018), and the main role is considered to be microglia (Colonna and Butovsky, 2017; Cable et al., 2020). Neuroinflammation is reportedly a complex process involving the coordination of different groups of glial cells, which act as a double-edged sword in this process, depending on the progression of the disease and the inflammatory microenvironment (Shabab et al., 2017; Yang and Zhou, 2019). The first attempt to visualize *in vivo* FPRs in mouse brains by positron emission tomography was undertaken by Lacivita et al. (2016). They found that FPRs are expressed in the microglia and mediate the chemotactic activity of Aβ peptides in AD, and they are thus involved in the chronic neuroinflammation process (Lacivita et al., 2016). The FPR mRNA is generally present in all regions of the brain, with higher expression in the brainstem and spinal cord (Ho et al., 2018). The biological significance of FPRs in the nerve cells and tissues has been elucidated through separate inflammatory signaling pathways.

Primarily, microglia cells monitor the physiological environment and act as the first line of defense when the brain suffers various injuries. For acute processes, such as ischemia and trauma, microglia cells maintain homeostasis by controlling the spread of neuroinflammation. Untimely chronic neuroinflammation causes neuronal damage, which is a prominent feature of progressive neurodegenerative diseases (Graeber, 2014; Leszek et al., 2016). The steady-state of the nervous system requires immunity surveillance and a suitable response from the microglia cells and peripheral immune cells via the release of pro-inflammatory cytokines and oxidative stress products (Abushouk et al., 2017). Animal experiments confirmed that the Th2-type cytokine IL-4 markedly inhibited TNF-α induced expression of mFPR2 in microglial cells by attenuating the activation of ERK and p38 MAPK, and that of NF-κB (Iribarren et al., 2005). Stimulation of brain mFPR2 with LXA1 or AnxA1 can inhibit microglial activation, thus diminishing neuroinflammation of several pathological conditions (Stama et al., 2017).

Astrocytes compose the most abundant subtype of neuroglial cells in the brain, and are intimately associated with brain homeostasis in the course of energy acquisition, neurotransmitters metabolism, and neuroinflammation (Dong and Benveniste, 2001). The activation of astrocytes in response to pathological conditions drive from the primary activation of microglia and cytokines such as IL-1 and IL-1β and subsequently release inflammatory cytokines. Reactive astrocytes divide into two phenotypes with strikingly opposite functions. On the one hand, they play potential beneficial role in synapse formation, neuronal development and proper propagation of action potentials (Liddelow and Barres, 2017). On the other hand, it is noteworthy that some pathological components, including oxidative stress, free saturated fatty acids, and lipopolysaccharide, upregulate reactive astrocytes to provoke Aβ-induced neuronal death and synaptic impairment (Sawikr et al., 2017). An increasing body of data has indicated that the FPRs exert their biological effects in neuroinflammation induced by astrocytes. Zhang et al. (2019) has carried out a trial in a mouse model of AD and reported that FPR2 deficiency facilitated activation of astrocytes and prognosis improvement. A previous U87 astrocytoma cell model study showed that the stimulation of FPR2 *in vitro* increased MAPK activities, which could be abrogated in the presence of an FPR2 antagonist. FPR2-induced ERK and JNK augment the expression of glial fibrillary acidic protein and IL-1α, which are correlated with reactive astrocytosis (Kam et al., 2007).

Several studies demonstrated that FPRs promote neural differentiation. Neural stem cells (NSCs), are used to study neurons and glial cells of stroke, along with the spinal cord, thus contributing to the rapid growth and repair of neural tissues. Activation of MAPK and PI3K/Akt pathways significantly increases NSC differentiation and decreases the number of apoptotic cells (Ishii et al., 2010). Wang et al. (2016) identified that mFPR1 and mFPR2 are expressed in NSCs, and mediate the migration of and promote the differentiation of NSCs to neurons. Further research conducted by this team proved that the self-renewal and neurogenesis of NSCs activated by mFPR2 signaling was regulated through the PI3K/Akt pathway and ROS production (Zhang et al., 2017). Moreover, mice lacking the mFPR1 gene show decreased expression of the second messengers, p38 MAPK and ERK, and reduced NF-κB translocation into the nucleus following traumatic brain injury (TBI) (Fusco et al., 2020). In addition, mFPR1 influences neurogenesis through regulation of the PI3K/Akt pathway (Le Belle et al., 2011; Fusco et al., 2020).

Axonal or Wallerian degeneration is a prominent early feature of most neurodegenerative diseases and nerve injuries. The activation of the Schwann cell (SC) phenotype after nerve injury is associated with inflammatory characteristics and inducing a microenvironment for axon regeneration (Stoll et al., 2002; Rotshenker, 2011; Dubovy et al., 2013, 2014). We speculate FPRs activate SCs, from which SAA originates after nerve injury and induce the cytokine monocyte chemoattractant protein-1 and macrophage inflammatory peptide-1α, which are synthesized from the SCs. The SAA may induce macrophage movement during Wallerian degeneration, which can be blocked by an FPR2-antagonist (Jang et al., 2012). Korimova et al. discovered that mitochondrial DAMPs released from axonal mitochondria modulate the outgrowth of cytoplasmic processes of the RT4 SC process in a dose- and time-dependent manner. Immunofluorescence staining also revealed that mFPR2 exists in the SCs and in the nerves distal to nerve injury (Korimova et al., 2018). In addition, mFPR2 is observed in growth cones, suggesting its involvement in axonal regeneration. Korimova et al. seconded this finding in a subsequent investigation (Korimova and Dubovy, 2020). Another analysis based on a facial nerve injury model corroborated the findings regarding the factors associated with remyelination involved in FPR. AnxA1 acts as the extracellular trigger for SC proliferation and migration, and activates FPR2 and the downstream adenosine 5′-monophosphate (AMP)-activated protein kinase (AMPK) inflammatory signaling cascade. AMPK phosphorylation induced by insulin-like growth factor-1 improves the mitochondrial function to drive axonal outgrowth, therefore promoting the SC proliferation and nerve regeneration (Xia et al., 2020).

**FIGURE 2:**
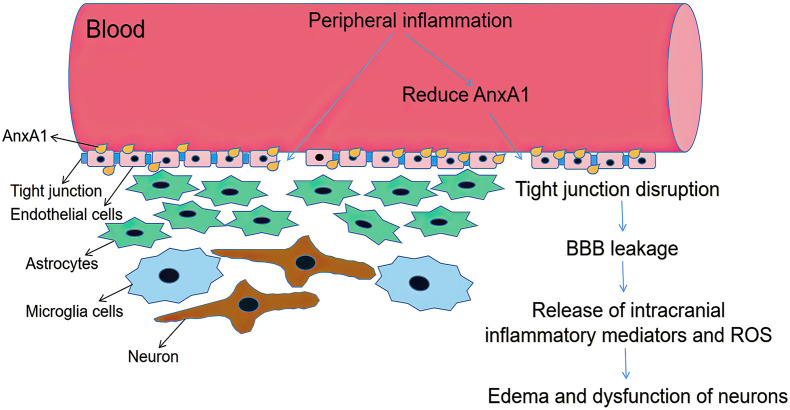
Schematic diagram illustrating BBB dysfunction. ANXA1 is an indispensable part of BBB, which mainly expressed by brain microvascular endothelial cells close to plasma membrane (Gussenhoven et al., 2019). Systemic inflammation can increase BBB permeability by reducing AnxA1, with increased paracellular permeability and decreased TJ protein expression. Decline in BBB integrity triggers glial activation thus release intracranial inflammatory mediators and ROS. Then it causes brain edema and dysfunction of neurons.

The BBB is the primary communication channel between systemic circulation and that in the brain, and its function is to prevent the destructive effects on the central nervous system (CNS) due to problems maintaining peripheral homeostasis during inflammation and metabolic diseases. Tight junctions (TJs) invade toxic substances and stop cells from passing through the vascular wall via the paracellular and transcellular pathways (Carman et al., 2007). Since the BBB is highly vulnerable to metabolic toxins, enhanced BBB-permeability can lead to edema, disruption of ionic homeostasis, and immune infiltration, causing neuronal dysregulation and depriving the BBB of its function (Daneman and Prat, 2015). The FPRs agonist AnxA1 is reportedly a critical component of the normal BBB. Exogenous human recombinant AnxA1 inhibited BBB disruption and intrusion of systemic inflammation into the brain through the Ras homolog gene family member A (RhoA) and rho-associated coiled-coil kinase (ROCK) inhibition, thereby improving functional outcomes in controlled cortical impact mice. The RhoA and the major downstream effector ROCK can enhance paracellular permeability by initiating the signal transduction pathway leading to the destabilization of the actin cytoskeleton during cell adhesion, migration, contraction, and proliferation (Terry et al., 2010). AnxA1 knockout mice exhibited a higher degree of neutrophil exosmosis associated with RhoA activity (Terry et al., 2010). The recombinant AnxA1 negatively regulated the RhoA/ROCK pathway and stabilized endothelial actin proteins and TJs; p38/MAPK phosphorylation increased significantly in response to cerebral inflammation-induced BBB disruption, whereas LXA4 treatment reduces inflammation through the FPR2/p38/MAPK signaling pathway (Guo et al., 2016). Thus, the signal cascade triggered by the combination of AnxA1 and FPR2 may be a target for the treatment of BBB dysfunction (Figure 2).

Of note, FPR1 and its variants FPR like 1 (FPRL1) serve as a critical component in angiogenesis, cell proliferation, and signaling pathways of neuroinflammation (Cattaneo et al., 2010). Pharmacological studies have recently confirmed that FPR1 exerted efficient effects on resisting neuroinflammation by restraining proinflammatory mediators, such as TNF-α and IL-1β, otherwise inducing IL-1RA and IL-10 expression (Calvello et al., 2021). FPR1 is expressed in neuronal tissues. Several signaling molecules, such as p38/MAPK, PKC, and PLC are required for FPR1 activation, which triggers NF-κB and STAT3 transcriptional factors. It has been described that FPR1 promotes neuronal differentiation of neural stem cells after TBI (Fusco et al., 2020). In the course of demyelination, FPR1 induces and maintains the glial cell activation thus is deemed as an important part of the innate immune in chronic degenerative diseases such as multiple sclerosis (Bihler et al., 2017). Most recent data showed that FPR1 deficiency alleviated brain edema and improved neurological outcomes in intracerebral hemorrhage (ICH) models (Li et al., 2021). Interestingly, FPRL1 also belongs to GPCRs. Expressed on both astrocytes and microglia, FPRL1 is generally thought to be involved in neuroinflammation. Using U87 astrocytoma cell model, Kwan and his colleagues demonstrated that FPRL1 gained access to promote calcium influx via switching store-operated channels (Kwan et al., 2008). Besides, FPRL1 has been shown to exert positive role in attenuating bacterial meningitis. Braun et al. evidenced that FPRL1 synergies with macrophage receptor with collagenous structure were essentially related in glial cell activation and antimicrobial peptides expression during resistance to meningitis (Braun et al., 2011).

## Role of Formyl Peptide Receptor in Neurological Disease Via Regulation of Inflammation

Due to the complexity, diversity, and multicellular process of neuroinflammation, it is firmly associated with a series of neurological diseases. FPRs are responsible for occurrence and prognosis of the disease.

### Formyl Peptide Receptors and Neurodegenerative Diseases

Alzheimer’s disease is the most common neurodegenerative disease and the single greatest cause of dementia. Aβ1-42 is an important component of the senile plaques in the brains of AD patients. *In vitro*, Aβ1-42 induces chemotaxis and release of neurotoxins by mice microglial cells activated by pro-inflammatory stimulants through mFPR2. Human FPR2 also contributes to the internalization of Aβ1-42 into the cytoplasmic compartment of macrophages, where Aβ1-42 formed fibrillary aggregates dependent on activation of MAPKs (Chen et al., 2007). In addition, Aβ1-42 induced ERK1/2 phosphorylation and a change of cyclic AMP accumulation in mice glial cells (Slowik et al., 2012).

It was postulated that FPR2 is both a mediator of neuroinflammation and a potential target of AD, even though FPR2 is relatively low-level in the brain microglia (Le et al., 2001a). The expression of FPR2 in microglia is significantly upregulated in the presence of inflammatory stimuli, including TNF-α and the agonists of toll-like receptor 2 and 4, especially fibrillar Aβ (fAβ). The microglia associated with amyloid plaques express high levels of FPR2 (Slowik et al., 2012). In one study, the microglia can induce FPR2 activation with an anti-inflammatory phenotype. FPR2 expression increases in plaque-associated microglia, which could affect the ability of microglia to engulf and decompose fAβ (Pan et al., 2011). AnxA1 reduces the Aβ level by increasing its enzymatic degradation by neprilysin in neuro 2a cells and by stimulating microglial Aβ phagocytosis, thus having protective effects on neuroinflammation in AD (Ries et al., 2016). Likewise, McArthur et al. evidenced that oligomer β-amyloid (oAβ) can inhibit the phagocytosis of fAβ by primary microglia, supporting the notion that oAβ may be more toxic than Aβ (McArthur et al., 2010). The oAβ-induced production of ROS may be associated with nicotinamide adenine dinucleotide phosphate (NADPH) oxidase activation, pentose phosphate pathway activation, and NADPH production, with changes that were reversed by mFPR2 ligand treatment. Microglial oAβ-stimulated ROS production could reportedly induce cell apoptosis, which could be prevented by mFPR2 ligand treatment, suggesting potential targets for therapeutic interventions (Wickstead et al., 2020). Thus, as a receptor for Aβ, FPR2 could remove Aβ, which has possible protective effects in the AD-brain. FPR2 could also induce glial cells to release pro-inflammatory factors after being activated by Aβ, indicating its harmful effect on the brain with reduced cognition and improved *tau* hyperphosphorylation (Zhang et al., 2019).

### Formyl Peptide Receptors and Neurogenic Tumor-Associated Inflammation

Gliomas are the most prevalent and malignant primary tumors in the brain and spinal cord. Malignant glioma cells can recognize growth-promoting signals and produce growth and angiogenesis factors for their proliferation. Glioma cells also express GPCRs, including FPRs, which transduce extracellular signals into intracellular effector pathways through the stimulation of heterotrimeric G proteins (Kranjc et al., 2019). This receptor superfamily includes GPCRs as classical chemoattractant receptors, inducing intracellular calcium mobilization, and activating MAPK/ERK, PI3K/Akt, and p38/MAPK signal transduction pathways (Snapkov et al., 2016) that modulate tumor cell proliferation and control cell migration and metastasis, regulate angiogenesis, and inhibit the immune cell infiltration into the tumor mass (Ambrosini and Aloisi, 2004). Stimulating the FPRs of the glioblastoma cells with fMLF can promote the release of vascular endothelial growth factor (VEGF) and IL-8. VEGF were recently shown to be involved in the growth and neovascularization of tumors. The removal of FPRs with specific siRNA eliminated the tumorigenicity of glioblastoma cells in nude mice (Yao et al., 2008). FPR expression is also responsible for increased motility of the human glioblastoma cells and the formation of highly invasive tumors (Huang et al., 2010). Moreover, the activation of FPRs leads to the transactivation of epidermal growth factor receptor (EGFR) in the glioblastoma cells. The mutual interaction between FPRs and EGFR aggravates the tumorigenicity of the glioblastoma cells. The fMLF rapidly induces EGFR phosphorylation at tyrosine residue 992, which contributes to the biological function of FPRs in the glioblastoma cells (Huang et al., 2007).

Annexin-1 is associated with tumor invasion, and its actions can be mediated by the receptor for FPRs (Tadei et al., 2018). In a recent study, a high expression of FPRs overlapped the AnxA1 immunolocalization in gliomas (Tadei et al., 2018). The expression of AnxA1 increased with the degree of malignancy, with overexpression in glioblastomas (Tadei et al., 2018). Moreover, the tumorigenicity of the glioblastoma cells was significantly reduced in AnxA1 knockout mice, while FPR1/AnxA1 double gene knockout mice were more effective in inhibiting tumor growth than AnxA1 knockout mice (Yang et al., 2011). Recent findings further demonstrated that AnxA1 regulates proliferation, migration, and invasion of the glioma cells via the PI3K/Akt signaling pathway (Wei et al., 2021).

### Formyl Peptide Receptors and Cerebrovascular Diseases

The interruption of blood supply to the brain and its adjoining tissues and blood vessels causes stroke, leading to death or permanent neurological impairment. The inflammatory state following ischemia-reperfusion (I/R) is reportedly a crucial contributing factor to these pathogenic processes. Post I/R inflammation activates endothelial cells, leukocytes, and platelets, leading to further microvascular dysfunction and subsequent tissue damage (Rodrigues and Granger, 2012). The MAPK signaling pathway, containing ERK1/2, JNK1/2, and p38, has been implicated in the regulation of cytokine expression and cell apoptosis after stroke (Meng et al., 2018). Since the brain is highly susceptible to I/R injury, recent studies have paid special attention to the involvement of FPRs in homeostasis following brain ischemic insult. FPR2 resolves inflammation by promoting non-phlogistic apoptosis, leukocyte phagocytosis, cell adhesion molecule shedding, and releasing anti-inflammatory cytokines in the tissues, thus being involved in ameliorating inflammatory storms in the early stages of reperfusion following stroke (Smith et al., 2015). FPR2 mediates its effects through agonists that have intrinsic mechanisms to mitigate cerebral I/R (Smith et al., 2015). Smith et al. (2015) examined leukocyte-endothelial interactions in the cerebral microvasculature during reperfusion using a murine bilateral common carotid artery occlusion model and its treatment with FPR agonists. They concluded that FPR2 modulated the I/R-associated vascular inflammatory responses. Similarly, activation of neutrophil FPR2/3 regulates the neutrophil-platelet aggregate formation in the brain (Vital et al., 2016). Further research by the same team indicated that distinct thrombo-inflammatory mice models were also significantly inhibited by the administration of Ac2-26. The mechanism includes suppressing thrombin-induced inside-out signaling events, such as Akt activation, intracellular calcium release, and Ras-associated protein 1 (Vital et al., 2016). In addition, ICH is a lethal stroke subtype. Recently animal experiments have confirmed that FPR2 acts as an effective remedy to ameliorate the neurological outcomes following ICH (Fan et al., 2019). Other study demonstrated that AnxA1 reduced microglia activation, remarkably relieved brain edema and improved short-term neurological function after ICH via inhibiting AnxA1/FPR2/p38 signaling pathway (Ding et al., 2020). These findings suggest that the AnxA1/FPR2 pathway represents a potential therapeutic and prophylactic target that may reduce the infarct volume and improve stroke outcomes without increasing the risk of ICH.

Another subtype of stroke, subarachnoid hemorrhage (SAH), causes high mortality and disability (Chen et al., 2014). It is believed that the early brain injury that occurs within 72 h of stroke onset heavily affects the prognosis of SAH (Xu et al., 2019). Inflammation is a crucial factor for an unfavorable prognosis resulting from BBB interruption, brain edema, and neuronal death after SAH, which involves p38/MAPK phosphorylation (Lai et al., 2020). An arachidonic acid metabolite, LXA4, binds to FPR2 and exerts potent anti-inflammatory actions by inhibiting pro-inflammatory cytokine production, suppressing neutrophil infiltration and enhancing the macrophage clearance (Wu et al., 2014). Following the onset of SAH, LXA4 expression decreases and the expression of pro-inflammatory factors and cytokines increases. The administration of LXA4 significantly ameliorated endothelial dysfunction, recovered microflow, and suppressed the inflammation and infiltration of neutrophils in rat models of SAH (Liu et al., 2019). Additionally, FPR2 expression increases after SAH, and adding LXA4 decreases the brain water content, which reduces BBB disruption, thereby improving neurological functions and recovering the learning and memory abilities (Guo et al., 2016).

Resolvin D1 is an endogenous ligand of FPR2, and its interactions reduce inflammation in stroke (Dufton and Perretti, 2010). RvD1 exerts a strong anti-inflammatory effect with the ability to reduce neutrophil infiltration and microglial pro-inflammatory activation via the activation of the FPR2/JNK/caspase3 pathway, leading to brain tissue restoration and improvement of neurological function (Cooray et al., 2013; Liu G. J. et al., 2021). RvD1 attenuated vasoconstriction and mitigated the poor prognosis caused by vasospasm in ischemia injury (Sansbury et al., 2020). RvD1 also attenuates hemoglobin-induced neuronal oxidative damage and apoptosis and possibly promotes anti-inflammatory polarization by regulating the IL-1 receptor-associated kinase 1/TNF receptor-associated factor 6 or MAPK signaling pathways (Liu G. J. et al., 2020). Therefore, RvD1 is expected to become useful in developing novel drugs for stroke treatment.

### Formyl Peptide Receptors and Spinal Cord Injury

Spinal cord injury (SCI) results from undue, uncontrolled spinal neuroinflammation. The damage induced by SCI is controlled by a biphasic pathophysiology, including the initial neural tissue damage and the following zone expansion of neural tissue injury, and exacerbation of neurological deficits (Consortium for Spinal Cord Medicine, 2008; Rowland et al., 2008; Yip and Malaspina, 2012). SCI induces an imbalance of edema and ischemia, and subsequently causes glial scar formation (Siddiqui et al., 2015), demyelination, and remyelination at the tissue level (Siddiqui et al., 2015). At the molecular level, SCI triggers the production of abnormal neurotrophic factors and their pro-peptides, cytokines, and chemokines (Gu et al., 2012; Ramesh et al., 2013; Richner et al., 2014). The activation of the MAPK and NF-κB signaling pathways following acute SCI is prone to upregulation of pro-inflammatory factors by the microglia (Liu G. et al., 2017; Liu Z. et al., 2020). Inhibition of the NF-κB signaling pathway in the spinal cord reduces secondary damage by inhibiting the microglia from expressing IL-6 and other pro-inflammatory cytokines (Zhou et al., 2016).

Recently, rapid investigations in SCI implicated the interaction of FPRs and their ligands in the inflammatory process following SCI onset, which can become new targets for the treatment of neuroinflammation. Resolvins and lipoxins are endogenous lipid mediators and ligands of FPRs contributing to the control of inflammatory responses during the immediate and long-term recovery from SAH (Serhan, 2005;Wang and Baker, 2018). They can also attenuate neuroinflammation and neuropathic pain (Svensson et al., 2007;Lima-Garcia et al., 2011). Studies on the neurophysiological mechanism based on the effects of aspirin-triggered RvD1 (AT-RvD1) on spinal nociceptive processing using *in vivo* electrophysiology characterized the acute effect of spinal administered AT-RvD1 on the induced spinal neuronal response, which prompt the biological effects of AT-RvD1 attributed to FPR2 (Meesawatsom et al., 2016). Concomitantly, LXA4 regulates the release of TNF-α from microglia through FPR2 to reduce neuropathic pain, indicating its endogenous anti-inflammatory and analgesic properties (Martini et al., 2016). Moreover, lipid mediators are involved in the resolution of synaptic plasticity (Ji et al., 2014). Recent evidence shows that nerve injury and its following inflammatory reaction induce the activation of the dorsal horn glial cells in the spine. LXA4 binds with FPR2 to attenuate NF-κB activation and blocks p38 and ERK phosphorylation in spinal astrocytes, thus mediating anti-nociception (Pannell et al., 2020). Thus, these results provide a novel insight into the regulation of peripheral and spinal sensitization and hyperalgesia by this lipid cascade and would be useful to elucidate the role of astrocytes and microglia in spinal nociceptive processing.

### Formyl Peptide Receptors and Other Nervous System Diseases

Epilepsy is characterized by hyperexcitability of neurons. Clinical and experimental evidence supports the hypothesis that neuroinflammation is a mechanism of epilepsy (Ambrogini et al., 2019). Increased IL-1β, IL-6, TNF-α, and their cognate receptors, and downstream effector molecules in the glial cells, neurons, and BBB cellular components contribute to the occurrence and recurrence of spontaneous epilepsy (Vezzani et al., 2011). FPR2 binds to AnxA1 to activate a series of signaling pathways, including intracellular calcium influx and activation of MAPKs, which contribute to synaptic excitability and cognitive impairment in the pathophysiology of epilepsy (Pernice et al., 2016). In the hippocampal neurons, the combination of FPR2 and Ac2-26 decreases the astrocyte marker glial fibrillary acidic protein, IL-1β, IL-6, growth-regulated α protein (GRO/KC), and ERK levels (Gimenes et al., 2019). FPR2 pathways associated with epileptogenic tissues detect the ability of a specific pro-resolving mediator, n-3 docosapentaenoic acid (DPA)-derived protectin D1 (PD_1*n–*3 DPA_), in suppressing the neuroinflammatory response during epileptogenesis (Frigerio et al., 2018). PD_1*n–*3 DPA_ is mediated by FPR2 and the chemerin receptor ChemR23/ERV1 to reduce the expression of pro-inflammatory molecules, regulating the migration of innate and adaptive immune cells to inflamed tissues, and restoring the BBB integrity (Cristante et al., 2013). These results may be significant to excavate the potential of FPR2 as a therapeutic target for epilepsy.

TBI is a brain damage obtained from external mechanical force leading to severe temporary or permanent impairment. The role of inflammation following TBI is obviously a key factor in aggravating neuronal damage. Immune cells such as glial cells, cerebrovascular endothelial cells and peripheral immune cells are recruited to the injury area to release excitotoxic cytokines and chemokines. ERK and p38/MAPK pathways have been confirmed to upregulate after TBI (Cristante et al., 2013). Accordingly, the absence of mFPR1 manifests the positive effect on trauma counteraction via restraining inflammation and oxidative stress. In the long term development after trauma, mFPR1 promotes neuronal differentiation of neural stem cells through PI3K/Akt pathway and reduces their differentiation into astrocytes (Fusco et al., 2020).

The pathophysiology of bacterial meningitis begins with host acquisition of opportunistic pathogens surviving nasopharyngeal colonization followed by systemic invasion, and subsequent development of high-grade bacteremia. The release of inflammatory mediators facilitates the pathogen to cross the BBB, triggering the peripheral immune system and cerebral edema (Gerber and Nau, 2010). The main effector cells of innate immune responses within the CNS are the glial cells, which have receptors including the toll-like receptors and FPRs, which can identify PAMPs and DAMPs (Migeotte et al., 2006). FPR deficiency is associated with increased bacterial burden and infiltration of the CNS by neutrophils and granulocytes, with distinct changes of the inflammatory immune reaction, resulting in higher mortality rates (Oldekamp et al., 2014). Recent research has indicated that the anti-inflammatory effect also applies to meningitis (Ruger et al., 2020). Another study using a mouse model of *Streptococcus suis*-induced meningitis, indicated that AnxA1 exerted anti-inflammatory effects via attenuating leukocyte infiltration, inflammatory mediator production, and astrocyte or microglial activation in the brain, along with decreasing neutrophil adherence to the endothelium through FPR2, thus inhibiting meningitis progression. Significantly, the study also revealed that AnxA1 decreases IL-6 expression and effect through the FPR2/p38/COX-2 pathway (Ni et al., 2021). These data highlight the potential therapeutic use of AnxA1 in bacterial meningitis.

Hypoxic ischemic encephalopathy (HIE) remains the primary cause of acute neonatal brain injury, with hypoxia-ischemia provoking an intravascular inflammatory cascade that is further augmented by the microglia and astrocyte response to cell damage in the brain parenchyma (Li et al., 2017). Thereafter, pro-inflammatory cytokines (TNF-α and IL-1β) promote neuronal apoptosis and necrosis, thus impairing neurological functions and inhibiting neurogenesis, which leads to secondary brain tissue injury (Ziemka-Nalecz et al., 2017). Activation of the p38/MAPK signaling pathway inhibits the learning, memory, and motor function of newborn rats with hypoxic-ischemic brain injury and promotes neuronal apoptosis in the hippocampal tissues (Zhu et al., 2020). A recent study found that FPRs play an important role in HIE. Activation of FPR2 with RvD1-attenuated neuroinflammation interacted with the Rac1/NOX2 signaling pathway, causing a decrease in the infarcted region and alleviating neurological deficits after HIE (Zhu et al., 2020). After hypoxic-ischemic injury, excessive glutamate release, free radical production, and an increase in BBB-permeability occur due to energy failure (Moretti et al., 2015). The process can be reversed by FPR2 activation. Moreover, AnxA1 restores endothelial resistance integrity following oxygen-glucose deprivation by FPR-dependence (Gussenhoven et al., 2019). Therefore, in the immature brain, AnxA1 targeting FPR2 has great potential in preventing BBB-loss and associated brain injury after HIE.

## Conclusion

**FIGURE 3:**
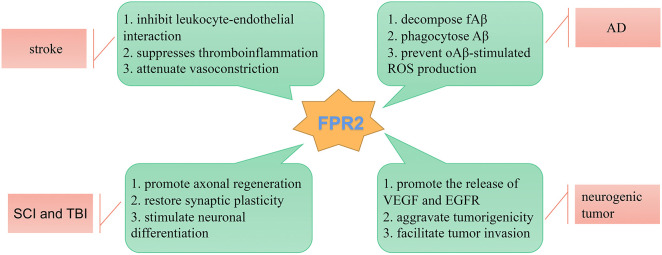
Schematic diagram illustrating the role of FPR2 in neurological diseases. FPR2 acts as a key molecule that mediates the active resolution of inflammation-related neurological diseases.

In recent years, much progress has been made in understanding the biological roles played by FPRs in the pathophysiological processes of neurological diseases. The FPR family has dual inflammatory functional properties, including pro-inflammatory and anti-inflammatory functions by triggering or inhibiting a series of GPCR intracellular kinase pathways, the activation of which can stimulate several signal transduction pathways depending on the ligands, its reaction microenvironment, and the cell type involved. It is beneficial to terminate transient inflammation through a proper decomposition process, which usually leads to a neuroprotective effect. Chronic inflammation leads to long-term homeostasis disease, permanent neurotoxicity, or neurodegenerative changes, and becomes the fundamental cause of CNS diseases. Neuroinflammation is central to several neurological diseases, including neurodegenerative diseases, stroke, neurogenic tumors, and SCI. Due to the complex and multifactorial nature of the structure and function within the nervous system, the accuracy and efficacy of therapeutic interventions for these neurological diseases remain unsatisfactory. Rapid developments in recent years have suggested that the specialized pro-resolving mediator, FPR2, acts as a key molecule that mediates the active resolution of inflammation in the CNS (Figure 3). AnxA1, LXA4, and RvD1 are common anti-inflammatory agonists, which bind with FPR2 to reduce the expression and activation of pro-inflammatory composition, govern the transport of immune cells to inflammatory tissues, and restore the integrity of the BBB. Thus, treatment with FPR2-modulation may be effective for neurological diseases. Recent studies are mainly limited to animal experiments, and the specificity of these mechanisms of FPR-mediated inflammation remains unclear. Future research should focus on the concrete and in-depth mechanisms of FPRs in inflammatory diseases of the human nervous system.

## Author Contributions

JZ, LL, JD, and JH performed the literature review and wrote the manuscript. AS and BT helped with the outline and article modification. All authors read and approved the final manuscript.

## Conflict of Interest

The authors declare that the research was conducted in the absence of any commercial or financial relationships that could be construed as a potential conflict of interest.

## Publisher’s Note

All claims expressed in this article are solely those of the authors and do not necessarily represent those of their affiliated organizations, or those of the publisher, the editors and the reviewers. Any product that may be evaluated in this article, or claim that may be made by its manufacturer, is not guaranteed or endorsed by the publisher.

## Abbreviations

A β: amyloid- β
AD: Alzheimer’s disease
AMP: adenosine 5’-monophosphate
AMPK: AMP-activated protein kinase
AnxA1: Annexin-1
Akt: protein kinase B
AT-RvD1: aspirin-triggered RvD1
BBB: blood brain barrier
CNS: central nervous system
DAMPs: damage-associated molecular patterns
EGFR: epidermal growth factor receptor
ENS: enteric nervous system
ERK: extracellular-regulated protein kinase
fA β: fibrillar A β
fMLF: N-formyl -leucine-phenylalanine
FPRs: formyl peptide receptors
GDP: guanosine diphosphate
GPCRs: G protein-coupled receptors
GTP: guanosine triphosphate
HIE: hypoxic ischemic encephalopathy
ICH: intracerebral hemorrhage
I/R: ischemia reperfusion
JNK: JUN-N-terminal protein kinase
LXA4: lipoxin A4
MAPK: mitogen-activated protein kinase
NADPH: nicotinamide adenine dinucleotide phosphate
NSCs: neural stem cells
oA β: oligomeric β -amyloid
PAMPs: pathogen-associated molecular patterns
PD_1n–3 DPA_: n-3 docosapentaenoic acid-derived protectin D1
PI3K: phosphoinositide-3-kinase
PLC: phospholipase C
PKC: protein kinase C
RhoA: Ras homolog gene family member A
ROCK: rho-associated coiled-coil kinase
ROS: reactive oxygen species
RvD1: resolvin D1
SAA: serum amyloid A
SAH: subarachnoid hemorrhage
SC: Schwann cell
SCIs: spinal cord injuries
TBI: traumatic brain injury
TJs: tight junctions
VEGF: vascular endothelial growth factor.
